# Early career psychiatrists in Europe during the COVID-19 pandemic: a cross-sectional study

**DOI:** 10.1017/S1092852925100734

**Published:** 2025-11-21

**Authors:** Mariana Pinto da Costa, Ozge Kilic, Egor Chumakov, Asilay Seker, Gaia Sampogna, Olga Kazakova, Jamila Ismayilova, Tove Mogren, Federico Mucci, Matilda Naesström, Franziska Baessler, Thomas Gargot, Victor Pereira-Sanchez, Diego Quattrone, Georgios Schoretsanitis, Ewelina Cichoń, Tomasz M. Gondek

**Affiliations:** 1 Institute of Psychiatry, Psychology & Neuroscience, King’s College London, London, UK; 2 Department of Psychiatry, Bezmialem Vakif University Medical Faculty, Istanbul, Türkiye; 3 Department of Psychiatry and Addiction, Saint-Petersburg State University, Saint-Petersburg, Russia; 4 Department of Psychiatry, University of Campania “L. Vanvitelli”, Italy; 5 Center for Epidemiology and Community Medicine, Sweden; 6 National Mental Health Center of the Ministry of Health of the Republic of Azerbaijan; 7 Department of Forensic Psychiatry, Sahlgrenska University Hospital, Sweden; 8 Department of Biotechnology, Chemistry and Pharmacy, University of Siena, Italy; 9 Department of Clinical Sciences/Psychiatry, Umeå University, Sweden; 10 Magdeburg University, Medical Faculty and Department of General, Internal and Psychosomatic Medicine, University Hospital Heidelberg, Germany; 11 Université de Tours, INSERM, Imaging Brain & Neuropsychiatry iBraiN U1253, 37032, Tours, France; 12 Department of Psychiatry, Columbia University, USA; 13 Social, Genetic and Developmental Psychiatry Centre, Institute of Psychiatry, Ps, UK; 14 Department of Psychiatry, Psychotherapy and Psychosomatics, Hospital of Psychiatry, University of Zurich, Switzerland; 15 Institute of Social Studies, University of Lower Silesia, Wroclaw, Poland

**Keywords:** COVID-19, telepsychiatry, psychiatry, education, training, healthcare workers

## Abstract

**Objective:**

We aimed to investigate the effects of the COVID-19 pandemic on the education and professional development, working conditions, and wellbeing of early career psychiatrists (ECPs) in Europe, as well as their attitudes to telepsychiatry.

**Methods:**

A questionnaire comprising 24 items was designed by the Early Career Psychiatrists Committee of the European Psychiatric Association. Data were collected online from June 2020 to September 2021. A series of multiple regression analyses were conducted to determine variables that could predict the dependent variables.

**Results:**

Data were obtained from 517 early career psychiatrists from 39 different countries. Men were more confident than women in their knowledge of COVID-19 symptoms and management, including in managing patients with a comorbidity of COVID-19 and a mental disorder. Providing specific recommendations during the COVID-19 pandemic, access to additional educational activities for ECPs, following COVID-19-related recommendations and access to protective equipment were the significant predictors of a higher confidence in managing patients with comorbidity of COVID-19 and mental disorders. The obligation to change the place of work predicted a decreased satisfaction with telepsychiatry as well as a decreased willingness to use telepsychiatry after the COVID-19 pandemic, while a diagnosis of COVID-19, having recommendations for telepsychiatry and access to dedicated platform for telemedicine were predictors of an increased satisfaction with telepsychiatry.

**Conclusions:**

The COVID-19 pandemic has affected ECPs in Europe to varying degrees. The results point to areas where decision-makers can improve the working conditions for ECPs.

## Introduction

Exposure to COVID-19 and pandemic-related public health measures like lockdowns has been linked to mental health impacts in the general population.^1^ Healthcare professionals constitute a subgroup at high risk for repercussions from the COVID-19 pandemic. This population is already at risk for burnout and poor mental health.^2^^,^^3^ Notably, physicians are especially at a high risk of developing adverse mental health outcomes, including symptoms of depression, anxiety, sleep problems, and increased fatigue.^2^^,^^4^^,^^5^ The high level of psychological distress among physicians may be linked to the COVID-19-related workload that exceeded the usual health care system capacity.^4^ This distress may have been intensified by the pervasive atmosphere of uncertainty, an influential factor for anxiety, changes in circadian rhythms, and the lack of knowledge regarding COVID-19, particularly in the first weeks after the outbreak.^4^^,^^6^^,^^7^ Importantly, healthcare professionals have faced substantial risks of contracting COVID-19, potentially subjecting them to the short- or long-term mental health consequences of COVID-19.^8^^,^^9^

Data regarding the impact of the COVID-19 on mental health professionals are scarce.^10^ Mental healthcare systems had to swiftly adapt following the first outbreak and during peaks of COVID-19, frequently struggling with resource shortages including protective equipment.^11^ Challenges encompassed change of workplace or using telepsychiatry without prior experience or training.^12^^–^^14^ Furthermore, several treatment options like brain stimulation treatments and electroconvulsive therapy became significantly constrained or even paused when COVID-19 started.^15^^–^^17^ Mental health professionals were relocated to join frontline management of COVID-19, where they were at higher risk of SARS-CoV-2 infection.^11^^,^^15^^,^^18^

Mental health professionals encountered levels of mental distress comparable to other medical specialties^19^ but also showed a noticeable prevalence of burnout and sleep problems, with younger individuals being at higher risk of burnout.^10^^,^^18^^,^^20^ A study in Saudi Arabia revealed a high incidence of burnout and depression symptoms among psychiatry trainees during the COVID-19 pandemic.^21^ As of now, the sole study investigating the preparedness and knowledge of early career psychiatrists during the pandemic has been conducted in Iran.^13^ The reports of early career psychiatrists working during the pandemic in different countries suggest that their experiences were similar to some extent, however, access to telepsychiatry and to personal protective equipment varied considerably between countries after the pandemic outbreak.^22^^,^^23^ It is suggested that the areas of psychiatric training that have been affected by the COVID-19 pandemic were quality of training, lack of diversified clinical exposure and practice, lack of research activities, use of technology for education, and effects on entrance and exit exams.^24^ Consequently, understanding the impact of the COVID-19 pandemic on ECPs in European countries is paramount.

The aim of the study has been to investigate the impact of the COVID-19 pandemic on education and professional development, working conditions, and wellbeing of early career psychiatrists in Europe, as well as their attitudes to telepsychiatry.

## Methods

### Study design

This was a cross-sectional international study among early-career psychiatrists. An online questionnaire (Supplementary Material) was developed by the Early Career Psychiatrists Committee (ECPC) of the European Psychiatric Association (EPA). The questionnaire included 24 items, and was structured in five sections: (1) socio-demographics, (2) COVID-19 knowledge and training, (3) workplace conditions, (4) wellbeing and support, and (5) telepsychiatry. Based on the survey, seven dependent variables were used in the analysis:

1. Confidence in knowledge of COVID-19 symptoms and management,
2. Confidence in managing patients with a comorbidity of COVID-19 and a mental disorder,
3. Adherence to physical distancing and other recommendations related to COVID-19 prevention at the workplace,
4. The impact of the COVID-19 pandemic on wellbeing,
5. The impact of supervisors and/or co-workers on wellbeing,
6. Satisfaction with the use of telepsychiatry during the pandemic,
7. Likeliness to use telemedicine after the pandemic.

### Data collection

Early career psychiatrists practicing in European countries were eligible to take part in this study. The EPA ECPC defines an “Early Career Psychiatrist” as a psychiatric trainee or a psychiatrist under 40 years of age or a psychiatrist less than 5 years after completing their specialty training (including both adult and child and adolescent psychiatrists).

In the beginning, participants confirmed that they met the study’s inclusion criteria and provided consent to participate in the study. The survey also included a question about professional status. Responses that did not fall into one of the following groups were excluded from this study: (1) trainee in general adult psychiatry, (2) trainee in child and adolescent psychiatry, (3) specialist in general adult psychiatry under 40 years of age or with less than 5 years of clinical practice after specialty, and (4) specialist in child and adolescent psychiatry under 40 years of age or with less than 5 years of clinical practice after specialty.

The questionnaire was administered online and information about the survey was distributed through websites, mailing lists, and social media of the EPA and the European Federation of Psychiatric Trainees (EFPT). Data were collected during the COVID-19 pandemic, from 16 June 2020 to 1 September 2021.

### Statistical analysis

A series of multiple regression analyses were conducted to determine variables that could predict the dependent variables, such as (1) confidence in the knowledge on COVID-19 symptoms and management; (2) confidence in managing patients with comorbidity of COVID-19 and mental disorders; (3) impact of COVID-19 on the wellbeing; (4) impact of supervisors and co-workers on wellbeing; (5) satisfaction with the use of telepsychiatry during the pandemic; and (6) willingness to use telemedicine after the pandemic. These variables were included as dependent variables in the separate regression models.

The selected characteristics and work-related conditions were included as predictors in the regression models: (1) gender (men or women); (2) the category of the country in which one is currently working according to the World Bank Classification (WBC) (high income or lowest income); (3) professional status (trainee or specialist); (4) professional areas (general adult psychiatry or child and adolescent psychiatry); (5) main place of work during the pandemic (in-patient or out-patient and other settings) (6) specific recommendations for ECPs during the pandemic (yes or no); (7) additional educational activities (courses, workshops, local conferences) on COVID-19 offered for ECPs (yes or no); (8) providing online obligatory local training and educational activities for ECPs (courses, workshops, local conferences) (yes or no); (9) extending the duration of training (yes or no); (10) reducing the duration of training (yes or no); (11) preventing from taking the specialist exam as planned (yes or no); (12) obligation by the authorities to change the place of work (yes or no); (13) following the recommendations related to COVID-19 prevention (eg physical distancing) at the workplace (yes or no); (14) personal protective equipment provided for the ECPs (yes or no); (15) access to free COVID-19 tests at place of work (yes or no); (16) diagnosis of COVID-19 (yes or no); (17) access to free psychological counselling (yes or no); (18) recommendations how to proceed with telepsychiatry (yes or no); and (19) access to dedicated closed platform for audiovisual communication in telemedicine (yes or no).

To detect potential collinearity or multicollinearity among the predicting variables, we calculated variance inflation factors (VIF). The higher value of VIF indicates a more problematic amount of collinearity between predictors.^25^ Values of more than 4 or 5 were considered as being moderate to high, with values of 10 or more being considered as very high.^26^^,^^27^ VIFs greater than 4 was also considered as indicator of possible problem with multicollinearity.^26^ In this study, the maximum value of VIF was 1.36 (see the VIFs in Table 6). These results allow us to assume no multicollinearity among the explanatory variables.

To run the series of multiple regressions, there were linearity as assessed by partial regression plots and a plot of studentized residuals against the predicted values. In each model, there was the independence of residuals, as assessed by the Durbin-Watson statistic. The Durbin-Watson statistic can range from 0 to 4. A value of approximately 2 indicates that there is no correlation between residuals.^28^ The values in this study were very close to 2 (1.869–2.116), so it can be accepted that there was independence of errors (residuals) (see Table 1).

**Table 1.** Participants’ Sociodemographic Data

	Men		Women		Total^a^	
	N	%	N	%	N	%
**Gender**	184	35.6%	330	63.8%	517	100.0%
**Professional status**						
Trainee in general adult psychiatry	84	45.7%	119	36.1%	205	39.7%
Trainee in child and adolescent psychiatry	18	9.8%	70	21.2%	88	17.0%
Specialist in general adult psychiatry under 40 years of age or with less than 5 years of clinical practice after specialty	73	39.7%	117	35.5%	191	36.9%
Specialist in child and adolescent psychiatry under 40 years of age or with less than 5 years of clinical practice after specialty	9	4.9%	24	7.3%	33	6.4%
**Main place of work during the COVID–19 pandemic**						
Inpatient psychiatric ward (or ER)	109	59.2%	152	46.1%	263	50.9%
Outpatient psychiatric clinic or day ward (or psychiatric rehabilitation facility or community mental health)	48	26.1%	122	37.0%	171	33.1%
Individual private psychiatric practice	16	8.7%	26	7.9%	42	8.1%
Inpatient or outpatient COVID–19 ward or clinic	7	3.8%	21	6.4%	28	5.4%
Other	4	2.2%	9	2.7%	13	2.5%
**Country where you currently work**						
Albania	1	0.5%	6	1.8%	7	1.4%
Armenia	2	1.1%	1	0.3%	3	0.6%
Austria	4	2.2%	14	4.2%	18	3.5%
Azerbaijan	0	0.0%	8	2.4%	8	1.5%
Belarus	2	1.1%	6	1.8%	8	1.5%
Belgium	3	1.6%	9	2.7%	12	2.3%
Croatia	3	1.6%	5	1.5%	8	1.5%
Cyprus	1	0.5%	2	0.6%	3	0.6%
Czech Republic	1	0.5%	0	0.0%	1	0.2%
Denmark	2	1.1%	5	1.5%	7	1.4%
Estonia	0	0.0%	3	0.9%	3	0.6%
Finland	0	0.0%	1	0.3%	1	0.2%
France	1	0.5%	7	2.1%	8	1.5%
Germany	7	3.8%	17	5.2%	24	4.6%
Greece	16	8.7%	25	7.6%	42	8.1%
Hungary	1	0.5%	5	1.5%	6	1.2%
Ireland	1	0.5%	1	0.3%	2	0.4%
Italy	15	8.2%	15	4.5%	31	6.0%
Kosovo	3	1.6%	0	0.0%	3	0.6%
Latvia	1	0.5%	3	0.9%	4	0.8%
Malta	3	1.6%	0	0.0%	3	0.6%
Moldova	2	1.1%	0	0.0%	2	0.4%
Montenegro	0	0.0%	2	0.6%	2	0.4%
Netherlands	3	1.6%	12	3.6%	15	2.9%
North Macedonia	0	0.0%	1	0.3%	2	0.4%
Norway	1	0.5%	2	0.6%	3	0.6%
Poland	23	12.5%	42	12.7%	65	12.6%
Portugal	3	1.6%	13	3.9%	16	3.1%
Romania	5	2.7%	12	3.6%	17	3.3%
Russia	26	14.1%	31	9.4%	57	11.0%
Serbia	1	0.5%	5	1.5%	6	1.2%
Slovakia	3	1.6%	1	0.3%	4	0.8%
Slovenia	0	0.0%	1	0.3%	1	0.2%
Spain	12	6.5%	13	3.9%	25	4.8%
Sweden	6	3.3%	16	4.8%	22	4.3%
Switzerland	13	7.1%	12	3.6%	25	4.8%
Turkey	11	6.0%	25	7.6%	36	7.0%
Ukraine	1	0.5%	0	0.0%	1	0.2%
United Kingdom	7	3.8%	9	2.7%	16	3.1%
**Country group according to World Bank classification**						
LMI – lower-middle income	1	0.5%	0	0.0%	1	0.2%
UMI – upper-middle income	53	28.8%	97	29.4%	151	29.2%
HI – high income	130	70.7%	233	70.6%	365	70.6%

a The “Total” column also includes those who identified their gender as non-binary or did not disclose their gender.

The bias-corrected and accelerated (BCa) bootstrap method using 2000 resamples was used as the recommended technique for computing confidence intervals for statistics that do not address the normality of the data.^29^^–^^31^

The variables measured on the ordinal scale (eg diagnosis of COVID-19) were recoded using the Dummy Coding method (coded variables: 0 = no, 1 = yes).^32^

All statistical analyses were done with SPSS version 29 for Windows.^33^ The level of significance was set at *p* < 0.05 in all statistical tests.

## Results

### Participants

**Figure 1.**
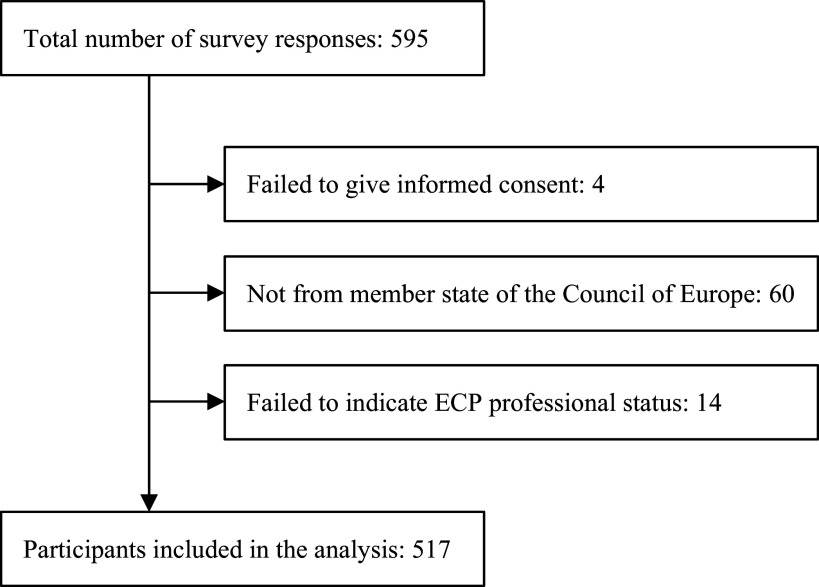
Flowchart.

A total of 595 people completed the survey, of which 517 met the study inclusion criteria. The reasons for excluding responses from the survey are presented in the flowchart (Figure 1).

### Socio-demographics

#### Gender

Women (n = 330) accounted for 63.8% of the study’s participants, men (n = 184) 35.6%. One person identified as non-binary (0.2%), and two did not want to specify their gender (0.4%).

#### Country of work

The included participants lived in 39 different European countries. In total, 365 participants were from high-income countries (HIC) based on the World Bank classification, 151 participants from upper-middle-income countries (UMIC), one participant from a lower-middle income country (LMIC). Currently, there are no low-income countries (LIC) among the European countries. For the purpose of comparisons between groups according to the World Bank classification, participants from UMI and LMI countries were combined into one group.

#### Professional status

Trainees in general adult psychiatry (n = 205) accounted for 39.7% of participants, while early-career specialists in general adult psychiatry (n = 191) accounted for 36.9%. Trainees in child and adolescent psychiatry (n = 88) represented 17.0% of participants and early-career specialists in child and adolescent psychiatry (n = 33) represented 6.4%.

#### Main place of work during the pandemic

ECPs in Europe worked in a variety of settings during the pandemic. The largest group (n = 263, 50.9%) worked in inpatient psychiatric units and/or in emergency psychiatric units. A total of 171 (33.1%) ECPs worked in a public outpatient mental health care facility (psychiatric clinics, day wards, psychiatric rehabilitation facilities, or community mental health teams). Forty-two (8.1%) participants worked in individual private psychiatric practice. There were 28 (5.4%) participants who worked in dedicated inpatient or outpatient COVID-19 wards or clinics, whereas 13 (2.5%) ECPs worked in other settings (eg university or prison).

The results of the following sections of the survey are presented in Tables 2–5.

**Table 2.** COVID-19 Knowledge and Training

	Men		Women		Total^a^	
	N	%	N	%	N	%
**How confident are you in your knowledge on COVID–19 symptoms and management?**						
Very confident	12	6,5%	23	7,0%	35	6,8%
Confident	94	51,1%	143	43,3%	239	46,2%
Neutral	65	35,3%	115	34,8%	181	35,0%
Unconfident	12	6,5%	42	12,7%	54	10,4%
Very unconfident	1	0,5%	7	2,1%	8	1,5%
**How confident are you in managing patients with a comorbidity of COVID–19 and a mental disorder?**						
Very confident	11	6,0%	6	1,8%	17	3,3%
Confident	62	33,7%	87	26,4%	150	29,0%
Neutral	80	43,5%	146	44,2%	228	44,1%
Unconfident	27	14,7%	79	23,9%	106	20,5%
Very unconfident	4	2,2%	12	3,6%	16	3,1%
**Have any specific recommendations for ECPs during the pandemic been introduced in your country? (multiple choice answer possible)**						
Yes, by the national/regional authorities	69	37,5%	96	29,1%	166	32,1%
Yes, by the National Psychiatric Association	39	21,2%	80	24,2%	120	23,2%
Yes, by other organizations	25	13,6%	39	11,8%	67	13,0%
Yes (total, recommendations introduced by any entity)	109	59,2%	169	51,2%	279	54,0%
No	75	40,8%	161	48,8%	238	46,0%
**Have additional educational activities (courses, workshops, local conferences) on COVID–19 been offered for ECPs in your country? (multiple choice answer possible)**						
Yes, general activities for all MDs	97	52,7%	150	45,5%	248	48,0%
Yes, specific activities dedicated to all psychiatrists	68	37,0%	91	27,6%	161	31,1%
Yes, specific activities dedicated to ECPs	12	6,5%	32	9,7%	45	8,7%
Yes (total)	127	69,0%	208	63,0%	335	64,8%
No	57	31,0%	122	37,0%	182	35,2%
**Have obligatory local training and educational activities for ECPs (courses, workshops, local conferences) been replaced with online activities?**						
Yes, all obligatory activities have been converted to online activities	75	40,8%	119	36,1%	194	37,5%
Yes, some but not all obligatory activities have been converted to online activities	89	48,4%	173	52,4%	263	50,9%
No, they have been carried out as usual (in person)	7	3,8%	6	1,8%	15	2,9%
No, they have been cancelled but no online alternative has been offered	13	7,1%	32	9,7%	45	8,7%
**Has COVID–19 pandemic affected the duration of your training? (multiple choice answer possible)**						
Yes, it extended the duration of my training	16	8,7%	41	12,4%	62	12,0%
Yes, it shortened the duration of my training	9	4,9%	29	8,8%	43	8,3%
Yes, it prevented me from taking the specialist exam as planned	5	2,7%	23	7,0%	34	6,6%
Yes (total: either changed the duration of my training or prevented me from taking exam as planned)	36	19,6%	89	27,0%	131	25,3%
No	93	50,5%	141	42,7%	234	45,3%
Not applicable (I have completed my training and exams before the COVID–19 outbreak)	55	29,9%	100	30,3%	152	29,4%

a The ‘Total’ column also includes those who identified their gender as non-binary or did not disclose their gender.

**Table 3.** Workplace Conditions

	Men		Women		Total	
	N	%	N	%	N	%
**Have you been obliged by the authorities to change the place of work because of the pandemic?**						
Yes	54	29,3%	84	25,5%	139^a^	26,9%
No	130	70,7%	246	74,5%	378^a^	73,1%
**The physical distancing and other recommendations related to the COVID–19 prevention were followed at my workplace**						
Strongly agree	43	23,4%	74	22,4%	117	22,6%
Agree	86	46,7%	179	54,2%	267	51,6%
Neither agree nor disagree	31	16,8%	41	12,4%	73	14,1%
Disagree	17	9,2%	24	7,3%	41	7,9%
Strongly disagree	4	2,2%	9	2,7%	13	2,5%
Not applicable (eg I work alone)	3	1,6%	3	0,9%	6	1,2%
**Have personal protective equipment been sufficiently provided for the ECPs in your workplace? (multiple choice answer possible)**						
Yes, they have been provided by the medical facility (hospital/clinic/university)	123	66,8%	214	64,8%	338	65,4%
Yes, they have been provided by the local or national authorities	38	20,7%	51	15,5%	90	17,4%
Yes, they have been provided by non-governmental organizations	18	9,8%	24	7,3%	44	8,5%
Yes, they have been provided by private entities or other individuals	34	18,5%	36	10,9%	71	13,7%
Yes (total)	144	78,3%	257	77,9%	403	77,9%
No, I had to buy or prepare the protective equipment myself	40	21,7%	73	22,1%	114	22,1%
**Did you have access to free COVID–19 tests at your place of work?**						
Yes	128	69,6%	217	65,8%	346	66,9%
No	56	30,4%	113	34,2%	171	33,1%
**Have you been tested for SARS-CoV–2?**						
Yes, at least one test positive	19	10,3%	33	10,0%	52	10,1%
Yes, all tests negative	105	57,1%	203	61,5%	311	60,2%
Yes, awaiting the results of my first test	1	0,5%	3	0,9%	4	0,8%
Yes (total)	125	67,9%	239	72,4%	367	71,0%
No	59	32,1%	91	27,6%	150	29,0%
**Have you been clinically diagnosed with COVID–19?**						
Yes, I have recovered	16	8,7%	32	9,7%	48	9,3%
Yes, I still have COVID–19	1	0,5%	1	0,3%	2	0,4%
Yes (total)	17	9,2%	33	10,0%	50	9,7%
No	167	90,8%	297	90,0%	467	90,3%
**Have you been quarantined?**						
Yes, due to clinical diagnosis of COVID–19	12	6,5%	19	5,8%	31	6,0%
Yes, due to positive test for COVID–19	7	3,8%	13	3,9%	20	3,9%
Yes, due to potential exposure to SARS-CoV–2 but not confirmed with test	37	20,1%	58	17,6%	97	18,8%
Yes, due to return from abroad	9	4,9%	14	4,2%	23	4,4%
Yes (total)	65	35,3%	104	31,5%	171	33,1%
No	119	64,7%	226	68,5%	346	66,9%

a The “Total” column also includes those who identified their gender as non-binary or did not disclose their gender.

**Table 4.** Well-being and Support

	Men		Women		Total^a^	
	N	%	N	%	N	%
**How did COVID–19 pandemic affect your well-being?**						
Very positively	3	1,6%	7	2,1%	10	1,9%
Rather positively	34	18,5%	52	15,8%	86	16,6%
No significant impact	49	26,6%	63	19,1%	113	21,9%
Rather negatively	86	46,7%	177	53,6%	265	51,3%
Very negatively	12	6,5%	31	9,4%	43	8,3%
**What was the impact of your supervisors and/or coworkers on your well-being?**						
Very positive	20	10,9%	19	5,8%	39	7,5%
Rather positive	43	23,4%	80	24,2%	123	23,8%
No significant impact	63	34,2%	101	30,6%	167	32,3%
Rather negative	39	21,2%	100	30,3%	139	26,9%
Very negative	12	6,5%	15	4,5%	27	5,2%
Not applicable	7	3,8%	15	4,5%	22	4,3%
**Did you have access to free psychological counselling?**						
Yes, provided/funded by the medical facility (hospital/clinic/university)	68	37,0%	76	23,0%	146	28,2%
Yes, provided/funded by the local or national authorities	19	10,3%	32	9,7%	51	9,9%
Yes, provided/funded by non-governmental organizations	16	8,7%	19	5,8%	35	6,8%
Yes, provided/funded by private entities or individual professionals	14	7,6%	27	8,2%	42	8,1%
Yes (total)	92	50,0%	130	39,4%	225	43,5%
No	92	50,0%	200	60,6%	292	56,5%

a The “Total” column also includes those who identified their gender as non-binary or did not disclose their gender.

**Table 5.** Telepsychiatry

	Men		Women		Total^a^	
	N	%	N	%	N	%
**Was telepsychiatry used in your country during the pandemic?**						
Yes, there are recommendations on how to proceed with telepsychiatry in my country	97	52,7%	146	44,2%	243	47,0%
Yes, however there are no recommendations on how to proceed with telepsychiatry in my country	76	41,3%	157	47,6%	236	45,6%
Yes (total)	173	94,0%	303	91,8%	479	92,6%
No, it is not legally approved in my country	11	6,0%	27	8,2%	38	7,4%
**What means of communication did you use for telepsychiatry? (multiple choice answer possible)**						
Dedicated closed platform for audiovisual communication in telemedicine	50	27,2%	71	21,5%	121	23,4%
General software for audiovisual communication (commonly used applications)	97	52,7%	138	41,8%	235	45,5%
Telephone or software for audio communication	84	45,7%	147	44,5%	233	45,1%
Chat, text messages or e-mail	38	20,7%	55	16,7%	93	18,0%
Various means of communication (mentioned above)	128	69,6%	195	59,1%	323	62,5%
Telephone or software for audio communication only	23	12,5%	54	16,4%	79	15,3%
Chat, text messages or e-mail only	7	3,8%	4	1,2%	11	2,1%
Not applicable	26	14,1%	77	23,3%	104	20,1%
**How satisfied are you with the use of telepsychiatry during the pandemic?**						
Very satisfied	22	12,0%	21	6,4%	43	8,3%
Rather satisfied	57	31,0%	63	19,1%	120	23,2%
Moderately satisfied	45	24,5%	99	30,0%	145	28,0%
Rather dissatisfied	25	13,6%	45	13,6%	70	13,5%
Very dissatisfied	9	4,9%	13	3,9%	23	4,4%
Not applicable, never used telepsychiatry	26	14,1%	89	27,0%	116	22,4%
**How likely are you to use telemedicine after the pandemic?**						
Extremely likely	33	17,9%	38	11,5%	71	13,7%
Likely	84	45,7%	102	30,9%	186	36,0%
Neutral	30	16,3%	86	26,1%	119	23,0%
Unlikely	21	11,4%	46	13,9%	67	13,0%
Extremely unlikely	8	4,3%	21	6,4%	29	5,6%
Not applicable (eg no legal conditions for telemedicine)	8	4,3%	37	11,2%	45	8,7%

a The “Total” column also includes those who identified their gender as non-binary or did not disclose their gender.

### COVID-19 knowledge and training

The majority of the study participants reported being “confident” (46.2%) or “neutral” (35%) in their knowledge on COVID-19 symptoms and management. However, with regard to confidence in managing patients with a comorbidity of COVID-19 and a mental disorder, the largest proportion of survey participants reported being neutral (44.1%), while confident (29%) or very confident (3.3%) responses were given by only a third of respondents in total. Of note is the difference in the frequency of “confident” and “very confident” responses between women (28.2% in total) and men (39.7% in total). More than half (54%) of ECPs received specific recommendations in their country during the pandemic and 64.8% had additional educational activities (courses, workshops, and local conferences) on COVID-19. For the vast majority of ECPs (88.4%), at least some of the obligatory local training and educational activities (courses, workshops, and local conferences) have been replaced with online activities. A quarter of respondents experienced either a change in the duration of their training or prevented them from taking the exam as planned during the pandemic period.

### Workplace conditions

More than a quarter of the participants (26.9%) have been obliged by the authorities to change the place of work because of the pandemic while almost three quarters of them (74.3%) “agreed” or “strongly agreed” that physical distancing and other recommendations related to the COVID-19 prevention were followed at their workplace. In total, 77.9% of the ECPs reported that personal protective equipment been sufficiently provided in their workplace. Two thirds of the participants (66.9%) had access to free COVID-19 tests at their place of work, while 71% have been tested for SARS-CoV-2. Almost one in ten (9.7%) of the ECPs have been clinically diagnosed with COVID-19, whereas one third (33.1%) have been quarantined.

### Wellbeing and support

The majority (59.2%) of the study participants have declared that COVID-19 pandemic affects their well-being “negatively” or “very negatively.” The assessment of the impact of coworkers and supervisors on the well-being of ECPs varied: 31.3% rated it as either “rather positive” or “very positive,” 32.1% as either “rather negative” or “very negative,” while 32.3% specified that they had no significant impact. Less than half (43.5%) of the study participants had access to free psychological counselling.

### Telepsychiatry

The vast majority (92.6%) of ECPs indicated that telepsychiatry was used in their countries during the pandemic. However, only about half of them had recommendations on how to proceed with telepsychiatry. The majority (62.5%) of the total number of participants in the study used various means of communication for telepsychiatry, with 17.4% of the participants only using non-video communication methods (“telephone or software for audio communication only” or “chat, text messages, or e-mail only”). Dedicated closed platforms for audiovisual communication in telemedicine were available to 23.4% of ECPs during the pandemic. Only 31.5% responded that they were “rather satisfied” or “very satisfied” with the use of telepsychiatry during the pandemic. Only 31.5% responded that they were “rather satisfied” or “very satisfied” with the use of telepsychiatry during the pandemic. However, half (49.7%) of the respondents assessed that it was “likely” or “very likely” that they were to use telemedicine after the pandemic.

#### Predictors of confidence in knowledge on COVID-19 symptoms and management among ECPs

The multiple regression was run to predict the confidence in the knowledge on COVID-19 symptoms and management among ECPs from the set of predictors. The multiple regression model statistically significantly predicted dependent variable (DV), *F*(19, 469) = 2.04, *p* = .006, *adj. R^2^* = .04. Five variables added statistically significantly to the prediction (*p* < .05).

The analysis indicated that men showed higher confidence in knowledge of COVID-19 symptoms and management than women when all variables were included in the model (*β* = .10, *t* = 2.23; *p* = .022; 95% BCa CI: 0.02–0.33). The results also indicated that the specialists (*β* = .11, *t* = 2.37; *p* = .018; 95% BCa CI: 0.03–0.35) were more confident than trainees. Moreover, reducing the duration of the training (*β* = .10, *t* = 2.13; *p* = .031; 95% BCa CI: 0.01–0.54), following COVID-19-related recommendations (*β* = .13, *t* = 2.90; *p* = .006; 95% BCa CI: 0.03–0.20) and access to a dedicated platform for telemedicine (*β* = .11, *t* = 2.34; *p* = .028; 95% BCa CI: 0.03–0.39) were the significant predictors of a higher confidence in knowledge on COVID-19 symptoms and management among ECPs (see Table 6).

**Table 6.** Predictors of Confidence in Knowledge on COVID-19 Symptoms and Management among ECPs (N = 508)

Model 1							
DV: Confidence in knowledge on COVID-19 symptoms and management							
Durbin-Watson value = 1.918							
Predictors	*B*	*SE B*	*β*	*T*	*p*	*95% BCa CI*	*VIF*
**Gender**							1.09
**Women (*ref.*)**							
**Men**	**0.18**	**0.08**	**0.10**	**2.23**	**.022**	**0.02–0.33**	
Country (acc. WBC)							1.36
UMI and LMI (*ref.*)							
HI	0.01	0.10	0.01	0.12	.908	−0.17 – 0.21	
**Professional status**							1.24
**Trainee (*ref.*)**							
**Specialist**	**0.19**	**0.08**	**0.11**	**2.37**	**.018**	**0.03–0.35**	
Professional areas							1.10
Adult psychiatry (*ref.*)							
Child and adolescent psychiatry	0.12	0.09	0.06	1.32	.215	−0.06 – 0.30	
Main place of work during the pandemic							1.20
Out-patient and others (*ref.*)							
In-patient	0.06	0.08	0.04	0.79	.457	−0.09 – 0.22	
Specific recommendations for ECPs during the pandemic							1.10
No (*ref.*)							
Yes	0.10	0.08	0.06	1.29	.204	−0.05 – 0.26	
Additional educational activities for ECPs							1.14
No (*ref.*)							
Yes	−0.07	0.09	−0.04	−0.88	.396	−0.25 – 0.09	
Providing online obligatory local training and educational activities for ECPs							1.08
No (*ref.*)							
Yes	−0.04	0.08	−0.02	−0.48	.653	−0.18 – 0.10	
Extending the duration of training							1.12
No (*ref.*)							
Yes	0.21	0.11	0.08	1.80	.059	−0.01 – 0.43	
**Reducing the duration of training**							1.07
**No (*ref.*)**							
**Yes**	**0.29**	**0.14**	**0.10**	**2.13**	**.031**	**0.01–0.54**	
Lack of possibility to take the exam on time							1.08
No (*ref.*)							
Yes	−0.13	0.14	−0.04	−0.87	.324	−0.42 – 0.11	
Obligation by the authorities to change the place of work							1.22
No (*ref.*)							
Yes	−0.15	0.09	−0.08	−1.69	.095	−0.33 – 0.03	
**Following COVID-related recommendations**							1.12
**No (ref.)**							
**Yes**	**0.12**	**0.04**	**0.13**	**2.90**	**.006**	**0.03–0.20**	
Providing protective equipment							1.23
No (ref.)							
Yes	−0.07	0.10	−0.03	−0.72	.487	−0.25 – 0.13	
Access to free COVID–19 tests at place of work							1.19
No (ref.)							
Yes	0.07	0.08	0.04	0.87	.352	−0.09 – 0.23	
Diagnosis of COVID–19							1.03
No (ref.)							
Yes	0.13	0.11	0.05	1.02	.266	−0.10 – 0.35	
Access to free psychological counselling							1.10
No (ref.)							
Yes	−0.03	0.08	−0.02	−0.36	.731	−0.18 – 0.14	
Recommendations for telepsychiatry							1.23
No (ref.)							
Yes	−0.02	0.08	−0.01	−0.29	.774	−0.19 – 0.14	
**Access to a dedicated platform for telemedicine**							1.16
**No (ref.)**							
**Yes**	**0.21**	**0.09**	**0.11**	**2.34**	**.028**	**0.03–0.39**	
**Model summary**	*R*	*R^2^*	*R^2^_adj_*		*F*(19,469)	*p*	
	0.271	0.074	0.038		2.04	= .006	

*Note.* Significant predictors are bold.

Abbreviation: WBC, World Bank Classification; HI, high-income; UMI, upper-middle-income; LMI, lower-middle income; 95% BCa CI, 95% bias-corrected and accelerated (BCa) bootstrap interval; *ref.*, reference category.

#### Predictors of confidence in managing patients with comorbidity of COVID-19 and mental disorders among ECPs

The multiple regression model statistically significantly predicted confidence in managing patients with comorbidity of COVID-19 and mental disorders among ECPs, *F*(19, 488) = 4.49, *p* < .001, *adj. R2* = .12. Thus, the model accounted for 12% of the variance of the dependent variable.

The analysis indicated that men showed higher confidence in managing patients with comorbidity of COVID-19 and mental disorders than women when all variables were included in the model (*β* = .12, *t* = 2.71; *p* = .009; 95% BCa CI: 0.06–0.35). The specialists (*β* = .12, *t* = 2.52; *p* = .018; 95% BCa CI: 0.05–0.36) were more confident than the trainees. Moreover, child and adolescent psychiatrists were less confident than general adult psychiatrists (*β* = −.09, *t* = −2.10; *p* = .030; 95% BCa CI: −0.35 to −0.03). Having specific recommendations (*β* = .12, *t* = 2.81; *p* = .003; 95% BCa CI: 0.07–0.36), access to additional educational activities for ECPs (*β* = .09, *t* = 2.12; *p* = .039; 95% BCa CI: 0.01–0.35), following COVID-19-related recommendations (*β* = .09, *t* = 2.09; *p* = .049; 95% BCa CI: 0.00–0.17) and access to a provided protective equipment (*β* = .12, *t* = 2.50; *p* = .016; 95% BCa CI: 0.05–0.43) were the significant predictors of a higher confidence in managing patients with comorbidity of COVID-19 and mental disorders among ECPs (see Table 7).

**Table 7.** Predictors of Confidence in Managing Patients with Comorbidity of COVID-19 and Mental Disorders Among ECPs (N = 508)

Model 2						
DV: Confidence in managing patients with comorbidity of COVID-19 and mental disorders						
Durbin-Watson value = 1.947						
Predictors	*B*	*SE B*	*β*	*t*	*p*	*95% BCa CI*
**Gender**						
**Women (*ref.*)**						
**Men**	**0.21**	**0.08**	**0.12**	**2.71**	**.009**	**0.06–0.35**
Country (acc. WBC)						
UMI and LMI (*ref.*)						
HI	−0.07	0.09	−0.04	−0.75	.469	−0.25 – 0.11
**Professional status**						
**Trainee (*ref.*)**						
**Specialist**	**0.20**	**0.08**	**0.12**	**2.52**	**.018**	**0.05–0.36**
**Professional areas**						
**Adult psychiatry (*ref.*)**						
**Child and adolescent psychiatry**	**−0.19**	**0.09**	**−0.09**	**−2.10**	**.030**	**−0.35 – −0.03**
Main place of work during the pandemic						
Out-patient and others (*ref.*)						
In-patient	0.00	0.08	0.00	0.04	.958	−0.15 – 0.15
**Specific recommendations for ECPs during the pandemic**						
**No (*ref.*)**						
**Yes**	**0.21**	**0.07**	**0.12**	**2.81**	**.003**	**0.07–0.36**
**Additional educational activities for ECPs**						
**No (*ref.*)**						
**Yes**	**0.18**	**0.08**	**0.09**	**2.12**	**.039**	**0.01–0.35**
Providing online obligatory local training and educational activities for ECPs						
No (*ref.*)						
Yes	0.03	0.08	0.02	0.35	.742	−0.13 – 0.18
Extending the duration of training						
No (*ref.*)						
Yes	0.05	0.12	0.02	0.45	.667	−0.16 – 0.26
Reducing the duration of training						
No (*ref.*)						
Yes	0.24	0.13	0.08	1.79	.054	−0.01 – 0.48
Lack of possibility to take the exam on time						
No (*ref.*)						
Yes	0.06	0.12	0.02	0.40	.608	−0.19 – 0.30
Obligation by the authorities to change the place of work						
No (*ref.*)						
Yes	−0.01	0.09	−0.01	−0.17	.863	−0.18 – 0.15
**Following COVID-19 related recommendations**						
**No (ref.)**						
**Yes**	**0.08**	**0.04**	**0.09**	**2.09**	**.049**	**0.00–0.17**
**Providing protective equipment**						
**No (ref.)**						
**Yes**	**0.23**	**0.10**	**0.12**	**2.50**	**.016**	**0.05–0.43**
Access to free COVID–19 tests at place of work						
No (ref.)						
Yes	0.14	0.08	0.08	1.68	.080	−0.02 – 0.31
Diagnosis of COVID–19						
No (ref.)						
Yes	0.13	0.13	0.04	1.04	.315	−0.13 – 0.36
Access to free psychological counselling						
No (ref.)						
Yes	0.01	0.08	0.01	0.17	.876	−0.14 – 0.16
Recommendations for telepsychiatry						
No (ref.)						
Yes	−0.07	0.08	−0.04	−0.87	.402	−0.24 – 0.08
**Access to dedicated platform for telemedicine**						
**No (ref.)**						
**Yes**	**0.23**	**0.10**	**0.12**	**2.57**	**.022**	**0.04–0.41**
**Model summary**	*R*	*R^2^*	*R^2^_adj_*	*F*(19,488)		*p*
	0.386	0.149	0.116	4.49		< .001

*Note.* Significant predictors are bold.

Abbreviation: WBC, World Bank Classification; HI, high-income; UMI, upper-middle-income; LMI, lower-middle income; 95% BCa CI, 95% bias-corrected and accelerated (BCa) bootstrap interval; *ref.*, reference category.

#### Predictors of the assessment of COVID-19 impact on the well-being among ECPs

Model 3 accounted for a significant amount of the variance in COVID-19 impact on the well-being among ECPs, *F*(19, 488) = 3.07, *p* < .001, adj. *R*^2^ = .072, and explained 7.2% of the variation in the dependent variable.

The analysis indicated that following COVID-19-related recommendations (*β* = .14, *t* = 2.98; *p* = .002; 95% BCa CI: 0.05–0.21) and access to the dedicated platform for telemedicine (*β* = .14, *t* = 3.05; *p* = .003; 95% BCa CI: 0.11–0.51) were the significant predictors of assessing COVID-19 as less negative for well-being (see Table 8).

**Table 8.** Predictors of Assessing COVID-19 Impact on the Well-being Among ECPs (N = 508)

Model 3						
DV: COVID-19 impact on the ECPs’ well-being						
Durbin-Watson value = 1.900						
Predictors	*B*	*SE B*	*β*	*t*	*p*	*95% BCa CI*
Gender						
Women (*ref.*)						
Men	0.15	0.09	0.07	1.68	.093	−0.02 – 0.31
Country (acc. WBC)						
UMI and LMI (*ref.*)						
HI	−0.01	0.10	0.00	−0.09	.929	−0.22 – 0.19
Professional status						
Trainee (*ref.*)						
Specialist	−0.06	0.09	−0.03	−0.72	.488	−0.25 – 0.11
Professional areas						
Adult psychiatry (*ref.*)						
Child and adolescent psychiatry	0.07	0.10	0.03	0.75	.482	−0.13 – 0.27
Main place of work during the pandemic						
Out-patient and others (*ref.*)						
In-patient	−0.08	0.09	−0.04	−0.87	.377	−0.24 – 0.08
Specific recommendations for ECPs during the pandemic						
No (*ref.*)						
Yes	−0.01	0.09	−0.01	−0.16	.877	−0.21 – 0.19
Additional educational activities for ECPs						
No (*ref.*)						
Yes	0.12	0.09	0.06	1.32	.204	−0.06 – 0.32
Providing online obligatory local training and educational activities for ECPs						
No (*ref.*)						
Yes	−0.05	0.09	−0.03	−0.59	.549	−0.22 – 0.12
Extending the duration of training						
No (*ref.*)						
Yes	−0.14	0.12	−0.05	−1.09	.268	−0.40 – 0.10
Reducing the duration of training						
No (*ref.*)						
Yes	0.01	0.14	0.00	0.09	.929	−0.26 – 0.29
Lack of possibility to take the exam on time						
No (*ref.*)						
Yes	0.25	0.19	0.07	1.48	.185	−0.14 – 0.63
Obligation by the authorities to change the place of work						
No (*ref.*)						
Yes	−0.18	0.10	−0.09	−1.87	.064	−0.37 – 0.00
**Following COVID-19 related recommendations**						
**No (ref.)**						
**Yes**	**0.13**	**0.04**	**0.14**	**2.98**	**.002**	**0.05–0.21**
Providing protective equipment						
No (ref.)						
Yes	0.11	0.11	0.05	1.07	.295	−0.11 – 0.34
Access to free COVID–19 tests at place of work						
No (ref.)						
Yes	−0.15	0.10	−0.07	−1.60	.119	−0.34 – 0.04
Diagnosis of COVID–19						
No (ref.)						
Yes	0.25	0.17	0.08	1.81	.154	−0.07 – 0.56
Access to free psychological counselling						
No (ref.)						
Yes	0.16	0.09	0.09	1.90	.063	−0.002 – 0.33
Recommendations for telepsychiatry						
No (ref.)						
Yes	0.07	0.09	0.04	0.74	.478	−0.11 – 0.25
**Access to dedicated platform for telemedicine**						
**No (ref.)**						
**Yes**	**0.31**	**0.10**	**0.14**	**3.05**	**.003**	**0.11–0.51**
**Model summary**	*R*	*R^2^*	*R^2^_adj_*	*F*(19,488)		*p*
	0.327	0.107	0.072	3.07		< .001

*Note.* Significant predictors are bold.

Abbreviation: WBC, World Bank Classification; HI, high-income; UMI, upper-middle-income; LMI, lower-middle income; 95% BCa CI, 95% bias-corrected and accelerated (BCa) bootstrap interval; *ref.*, reference category.

#### Predictors of the assessment of supervisors’ and co-workers’ impact on the well-being of ECPs

Regarding the assessment of supervisors’ and co-workers’ impact on the well-being of ECPs, the multiple regression indicates that the set of selected predictors contributed significantly to the regression model, *F*(19, 469) = 4.21, *p* < .001, *adj R2* = .111) and accounted for 11.1% of the relevance of supervisors and co-workers for ECPs’ well-being.

In particular, the analysis indicated that additional educational activities for ECPs (*β* = .09, *t* = 2.03; *p* = .049; 95% BCa CI: 0.01–0.41), lack of possibility to take the exam on time (*β* = .09, *t* = 1.99; *p* = .023; 95% BCa CI: 0.05–0.69), following COVID-related recommendations (*β* = .25, *t* = 5.55; *p* < .001; 95% BCa CI: 0.18–0.38), diagnosis of COVID-19 (*β* = .08, *t* = 1.96; *p* = .035; 95% BCa CI: 0.03–0.56), and access to free psychological counselling (*β* = .16, *t* = 3.65; *p* < .001; 95% BCa CI: 0.15–0.52) predict the assessment of the impact of supervisors’ and co-workers’ on ECPs’ well-being as more positive (see Table 9).

**Table 9.** Predictors of the Supervisors’ and Co-workers’ Impact on the Well-being of ECPs (N = 489)

Model 4						
DV: Supervisors’ and co-workers’ impact on the ECPs’ well-being						
Durbin-Watson value = 1.917						
Predictors	*B*	*SE B*	*β*	*t*	*p*	*95% BCa CI*
Gender						
Women (*ref.*)						
Men	0.14	0.10	0.07	1.47	.174	−0.05 – 0.35
Country (acc. WBC)						
UMI and LMI (*ref.*)						
HI	−0.12	0.11	−0.05	−1.07	.281	−0.36 – 0.11
Professional status						
Trainee (*ref.*)						
Specialist	−0.15	0.10	−0.07	−1.49	.138	−0.35 – 0.04
Professional areas						
Adult psychiatry (*ref.*)						
Child and adolescent psychiatry	0.13	0.11	0.05	1.21	.235	−0.08 – 0.34
Main place of work during the pandemic						
Out-patient and others (*ref.*)						
In-patient	0.07	0.10	0.04	0.77	.440	−0.12 – 0.27
Specific recommendations for ECPs during the pandemic						
No (*ref.*)						
Yes	−0.11	0.10	−0.05	−1.19	.252	−0.28 – 0.07
**Additional educational activities for ECPs**						
**No (*ref.*)**						
**Yes**	**0.21**	**0.10**	**0.09**	**2.03**	**.049**	**0.01–0.41**
Providing online obligatory local training and educational activities for ECPs						
No (*ref.*)						
Yes	−0.01	0.10	−0.01	−0.11	.915	−0.20 – 0.16
Extending the duration of training						
No (*ref.*)						
Yes	−0.04	0.14	−0.01	−0.27	.769	−0.33 – 0.22
Reducing the duration of training						
No (*ref.*)						
Yes	−0.19	0.18	−0.05	−1.16	.293	−0.54 – 0.16
**Lack of possibility to take the exam on time**						
**No (*ref.*)**						
**Yes**	**0.37**	**0.17**	**0.09**	**1.99**	**.023**	**0.05–0.69**
Obligation by the authorities to change the place of work						
No (*ref.*)						
Yes	−0.15	0.10	−0.06	−1.34	.164	−0.35 – 0.06
**Following COVID-19 related recommendations**						
**No (ref.)**						
**Yes**	**0.28**	**0.05**	**0.25**	**5.55**	**.000**	**0.18–0.38**
Providing protective equipment						
No (ref.)						
Yes	−0.02	0.12	−0.01	−0.21	.839	−0.26 – 0.20
Access to free COVID–19 tests at place of work						
No (ref.)						
Yes	−0.18	0.10	−0.08	−1.71	.082	−0.37 – 0.01
**Diagnosis of COVID–19**						
**No (ref.)**						
**Yes**	**0.29**	**0.14**	**0.08**	**1.96**	**.035**	**0.03–0.56**
**Access to free psychological counselling**						
**No (ref.)**						
**Yes**	**0.34**	**0.10**	**0.16**	**3.65**	**.001**	**0.15–0.52**
Recommendations for telepsychiatry						
No (ref.)						
Yes	0.00	0.10	0.00	0.01	.985	−0.20 – 0.21
Access to dedicated platform for telemedicine						
No (ref.)						
Yes	0.17	0.12	0.07	1.56	.131	−0.06 – 0.43
**Model summary**	*R*	*R^2^*	*R^2^_adj_*	*F*(19,469)		*p*
	0.382	0.146	0.111	4.21		< .001

*Note.* Significant predictors are bold.

Abbreviation: WBC, World Bank Classification; HI, high-income; UMI, upper-middle-income; LMI, lower-middle income; 95% BCa CI, 95% bias-corrected and accelerated (BCa) bootstrap interval; *ref.*, reference category.

#### Predictors of satisfaction with the use of telepsychiatry during the COVID-19 pandemic among ECPs

The analysis indicated that the model, including the set of predictors, had explained a 15.1% variation in satisfaction with the use of telepsychiatry during the COVID-19 pandemic, *F*(19, 365) = 4.69; *p* < .001, *adj. R^2^* = 0.151).

It turned out that the obligation by the authorities to change the place of work (*β* = −0.11, *t* = −2.07, *p* = .035; 95% BCa CI: −0.46 – −0.02) was a predictor of decreased satisfaction with telepsychiatry. Whereas, diagnosis of COVID-19 (*β* = 0.11, *t* = 2.24, *p* =. 022; 95% BCa CI: 0.04–0.73), having recommendations for telepsychiatry (*β* = 0.20, *t* = 4.02, *p* <. 001; 95% BCa CI: 0.22–0.63) as well as access to dedicated platform for telemedicine (*β* = 0.21, *t* = 4.09, *p* <. 001; 95% BCa CI: 0.26–0.67) were related to increased satisfaction with telepsychiatry (see Table 10).

**Table 10.** Predictors of Satisfaction with the Use of Telepsychiatry During the COVID-19 pandemic Among ECPs (N = 395)

Model 5						
DV: Satisfaction with the use of telepsychiatry						
Durbin-Watson value = 2.116						
Predictors	*B*	*SE B*	*β*	*T*	*p*	*95% BCa CI*
Gender						
Women (*ref.*)						
Men	0.15	0.10	0.07	1.51	.126	−0.05 – 0.36
Country (acc. WBC)						
UMI and LMI (*ref.*)						
HI	−0.27	0.14	−0.11	−2.10	.060	−0.54 – 0.01
Professional status						
Trainee (*ref.*)						
Specialist	0.14	0.11	0.07	1.29	.212	−0.09 – 0.38
Professional areas						
Adult psychiatry (*ref.*)						
Child and adolescent psychiatry	0.00	0.12	0.00	−0.03	.969	−0.24 – 0.23
Main place of work during the pandemic						
Out-patient and others (*ref.*)						
In-patient	−0.02	0.11	−0.01	−0.22	.832	−0.24 – 0.21
Specific recommendations for ECPs during the pandemic						
No (*ref.*)						
Yes	−0.04	0.10	−0.02	−0.41	.669	−0.24 – 0.16
Additional educational activities for ECPs						
No (*ref.*)						
Yes	0.22	0.11	0.10	1.97	.056	−0.01 – 0.45
Providing online obligatory local training and educational activities for ECPs						
No (*ref.*)						
Yes	−0.03	0.10	−0.02	−0.32	.754	−0.23 – 0.15
Extending the duration of training						
No (*ref.*)						
Yes	0.30	0.15	0.09	1.78	.047	−0.01 – 0.60
Reducing the duration of training						
No (*ref.*)						
Yes	0.24	0.17	0.07	1.35	.148	−0.11 – 0.58
Lack of possibility to take the exam on time						
No (*ref.*)						
Yes	−0.21	0.24	−0.05	−0.93	.359	−0.67 – 0.25
**Obligation by the authorities to change the place of work**						
**No (*ref.*)**						
**Yes**	**−0.24**	**0.11**	**−0.11**	**−2.07**	**.035**	**−0.46 – −0.02**
Following COVID-related recommendations						
No (ref.)						
Yes	0.09	0.06	0.09	1.72	.116	–0.02 – 0.21
Providing protective equipment						
No (ref.)						
Yes	−0.11	0.13	−0.04	−0.82	.425	−0.36 – 0.17
Access to free COVID–19 tests at place of work						
No (ref.)						
Yes	0.04	0.11	0.02	0.33	.744	−0.18 – 0.25
**Diagnosis of COVID–19**						
**No (ref.)**						
**Yes**	**0.40**	**0.17**	**0.11**	**2.24**	**.022**	**0.04–0.73**
Access to free psychological counselling						
No (ref.)						
Yes	0.17	0.10	0.08	1.70	.085	−0.03 – 0.37
**Recommendations for telepsychiatry**						
**No (ref.)**						
**Yes**	**0.42**	**0.10**	**0.20**	**4.02**	**.000**	**0.22–0.63**
**Access to dedicated platform for telemedicine**						
**No (ref.)**						
**Yes**	**0.47**	**0.11**	**0.21**	**4.09**	**.000**	**0.26–0.67**
**Model summary**	*R*	*R^2^*	*R^2^_adj_*	*F*(19,375)		*p*
	0.438	0.192	0.151	4.69		< .001

*Note.* Significant predictors are bold.

Abbreviation: WBC, World Bank Classification; HI, high-income; UMI, upper-middle-income; LMI, lower-middle income; 95% BCa CI, 95% bias-corrected and accelerated (BCa) bootstrap interval; *ref.*, reference category.

#### Predictors of willingness to use telemedicine after the COVID-19 pandemic among ECPs

Model 6 accounted for a significant amount (10.6%) of the variance in the willingness to use telemedicine after the COVID-19 pandemic among ECPs, *F*(19, 443) = 3.90, *p* < .001, adj. *R*^2^ = .106.

The analysis indicated that men showed a greater willingness to use telemedicine after the COVID-19 pandemic than women (*β* = 0.11, *t* = 2.31; *p* = .020; 95% BCa CI: 0.03–0.45). Moreover, additional educational activities for ECPs (*β* = 0.11, *t* = 2.33; *p* = .020; 95% BCa CI: 0.05–0.46), having recommendations for telepsychiatry (*β* =0.11, *t* = 2.20; *p* = .022; 95% BCa CI: 0.04–0.44), and access to the dedicated platform for telemedicine (*β* = 0.23, *t* = 4.84; *p* < .001; 95% BCa CI: 0.37–0.79) were the significant predictors of the greater willingness to use telemedicine after the COVID-19 pandemic, whereas the obligation by the authorities to change the place of work (*β* = −0.11, *t* = −2.32, *p* = .017; 95% BCa CI: −0.50 – −0.07) predict decreased willingness (see Table 11).

**Table 11.** Predictors of Willingness to Use Telemedicine after the Pandemic Among ECPs (N = 463)

Model 6						
DV: Willingness to use telemedicine after pandemic						
Durbin-Watson value = 1.869						
Predictors	*B*	*SE B*	*Β*	*T*	*p*	*95% BCa CI*
**Gender**						
**Women (*ref.*)**						
**Men**	**0.24**	**0.11**	**0.11**	**2.31**	**.020**	**0.03–0.45**
Country (acc. WBC)						
UMI and LMI (*ref.*)						
HI	−0.15	0.12	−0.06	−1.20	.212	−0.38 – 0.07
Professional status						
Trainee (*ref.*)						
Specialist	0.12	0.11	0.05	1.05	.296	−0.10 – 0.36
Professional areas						
Adult psychiatry (*ref.*)						
Child and adolescent psychiatry	−0.14	0.12	−0.06	−1.20	.229	−0.37 – 0.09
Main place of work during the pandemic						
Out-patient and others (*ref.*)						
In-patient	0.03	0.11	0.01	0.29	.773	−0.18 – 0.25
Specific recommendations for ECPs during the pandemic						
No (*ref.*)						
Yes	−0.02	0.11	−0.01	−0.23	.824	−0.23 – 0.18
**Additional educational activities for ECPs**						
**No (*ref.*)**						
**Yes**	**0.26**	**0.11**	**0.11**	**2.33**	**.020**	**0.05–0.46**
Providing online obligatory local training and educational activities for ECPs						
No (*ref.*)						
Yes	−0.15	0.10	−0.07	−1.44	.156	−0.36 – 0.04
Extending the duration of training						
No (*ref.*)						
Yes	0.16	0.16	0.04	0.96	.304	−0.14 – 0.47
Reducing the duration of training						
No (*ref.*)						
Yes	0.03	0.16	0.01	0.18	.836	−0.28 – 0.34
Lack of possibility to take the exam on time						
No (*ref.*)						
Yes	−0.18	0.25	−0.04	−0.87	.458	−0.67 – 0.33
**Obligation by the authorities to change the place of work**						
**No (*ref.*)**						
**Yes**	**−0.28**	**0.11**	**−0.11**	**−2.32**	**.017**	**−0.50 – −0.07**
Following COVID-19 related recommendations						
No (ref.)						
Yes	–0.07	0.05	−0.06	−1.24	.225	−0.17 – 0.04
Providing protective equipment						
No (ref.)						
Yes	0.00	0.13	0.00	0.02	.983	−0.24 – 0.25
Access to free COVID–19 tests at place of work						
No (ref.)						
Yes	−0.04	0.12	−0.02	−0.32	.746	−0.28 – 0.22
Diagnosis of COVID–19						
No (ref.)						
Yes	0.23	0.15	0.06	1.40	.115	−0.06 – 0.51
Access to free psychological counselling						
No (ref.)						
Yes	0.15	0.11	0.07	1.50	.166	−0.06 – 0.37
**Recommendations for telepsychiatry**						
**No (ref.)**						
**Yes**	**0.23**	**0.10**	**0.11**	**2.20**	**.022**	**0.04–0.44**
**Access to dedicated platform for telemedicine**						
**No (ref.)**						
**Yes**	**0.57**	**0.11**	**0.23**	**4.84**	**.000**	**0.37–0.79**
**Model summary**	*R*	*R^2^*	*R^2^_adj_*	*F*(19,443)		*p*
	0.378	0.143	0.106	3.90		< .001

*Note.* Significant predictors are bold.

Abbreviation: WBC, World Bank Classification; HI, high-income; UMI, upper-middle-income; LMI, lower-middle income; 95% BCa CI, 95% bias-corrected and accelerated (BCa) bootstrap interval; *ref*, reference category.

## Discussion

ECPs coped differently during the COVID-19 pandemic, contingent upon their work settings and conditions. A previously published article from Iran used the same questionnaire translated into Persian, which allows a direct comparison of the results with our study.^13^ In Europe overall we found slightly more participants being confident about their knowledge than in Iran. On the other hand, Iranian ECPs tended to be more confident in managing patients with a comorbidity of COVID-19 and a mental disorder.

The extent of COVID-19 knowledge may be associated with one’s undergraduate education. In Germany a study with medical students reported that 64% were dissatisfied with teaching on COVID-19 disease and 73% with teaching on COVID-19 vaccines.^34^

The results, which reveal a higher self-assessment of their knowledge and competence among men compared to women could be attributed to a general tendency for men to overestimate their competence, as described in the literature.^35^^,^^36^ A study comprising healthcare workers and students from the Gulf Cooperation Council region demonstrated similar COVID-19 knowledge levels between genders, albeit with slightly higher raw scores for men.^37^ On the other hand, a review on healthcare professional experiences of women during outbreaks suggests that disparities affecting women include a higher risk of pathogen exposure and infection, poorer access to personal protective equipment, increased workload, and higher prevalence of mental health problems.^38^

Compared to ECPs from Iran, a smaller proportion of ECPs working in Europe had specific recommendations introduced during the COVID-19 pandemic and were offered additional educational activities on COVID-19. Similarly, less participants from Europe had some or all of their obligatory local training and educational activities replaced with online activities than in Iran. However, the pandemic has affected the duration of the training of over one third of ECPs from Iran, while in European countries the duration of training of only a quarter of participants has been altered.^13^

Much more respondents from Europe, compared to Iran, agreed that the physical distancing and other recommendations related to COVID-19 prevention were followed at their workplace. The majority of participants in both Iran and Europe were sufficiently provided with personal protective equipment in their workplace. However, much more European ECPs had access to free COVID-19 tests in their place of work and were tested for SARS-CoV-2.^13^

A study on psychiatric trainees from 22 countries showed burnout is a common phenomenon among early career psychiatrists.^39^ Severe burnout was experienced by over one third of trainees and it was significantly associated with long working hours, lack of supervision and not having regular time to rest. In a study from Saudi Arabia on a group of 150 psychiatry trainees, over a quarter showed symptoms of burnout, and the same proportion had depressive symptoms.^21^ Those who suffered from burnout were almost nine times more likely to show symptoms of depression. Trainees who were women, who were in the first 2 years of training or who have received any mental health help in the previous 2 years were more likely to show depressive symptoms. In the previously mentioned study,^13^ the majority of Iranian participants described that the pandemic had negatively affected their wellbeing. A similar percentage of ECPs in Europe were affected negatively, albeit they tended to be affected to a lesser extent. Interestingly, twice as many European participants reported negative effects of their supervisors and/or co-workers on their well-being in comparison to those from Iran. Access to free psychological counseling was more available for ECPs in Iran than to those in Europe. More participants from Europe were obliged by the authorities to change their place of work because of the pandemic. There was a substantial difference in the proportion of people who were diagnosed with COVID-19 between European countries (10%) and Iran (62%). Similarly, 70% of Iranian ECPs were quarantined, as compared with 33% of quarantined ECPs in Europe.

ECPs from various countries agreed during the pandemic that telepsychiatry should be used more widely, particularly in rural and remote areas.^22^ A similar total percentage of participants used telepsychiatry in Iran and in European countries; however, more Iranian ECPs declared they did not have access to any recommendations on how to proceed with telepsychiatry consultations. Despite that, participants from Iran were more satisfied with telepsychiatry than their European colleagues and reported to be much more likely to use telemedicine after the pandemic.^13^

## Limitations of the study

The data collection period was long, and the sample size was large (n >500 people). The course of the COVID-19 pandemic was variable, with periods of waves of increasing incidence, the severity of which also varied between countries, which may have influenced the responses given. The individual situation of respondents may have changed with the course of the pandemic, while answers to the survey were given once. The questionnaire was administered online and it was not possible to calculate the response rate. There could have been a possible bias related to the participation of those individuals who had a greater interest in the survey topic, including potentially those for whom the COVID-19 pandemic represented a greater burden. On the other hand, difficulties related to working conditions during the pandemic may have meant that fewer participants with a greater workload during this period found time to participate in the study.

Due to the large differences in the number of responses between countries, it was not possible to carry out a comparative analysis between them. Finally, the questionnaire used was developed in English for the purpose of this study. This might have limited non-English speakers to participate in the study, and the reliability of the answers provided may have depended on the degree of English proficiency.

Due to these limitations, the generalizability of the results obtained is to a certain extent limited.

## Recommendations for changes in ECPs training and working conditions

We have identified independent factors that may be associated with better outcomes in terms of ECPs’ confidence in their knowledge and competencies and their adherence to pandemic-prevention guidelines, as well as with better well-being outcomes and satisfaction with telepsychiatry use. The majority of these factors are modifiable conditions linked to education and working arrangements, which can be improved. These conditions include (1) introduction of specific recommendations for ECPs during the pandemic, (2) additional educational activities for ECPs focused on the outbreak, (3) adequate provision of personal protective equipment in the workplace, (4) access to free psychological counselling, (5) no obligation by the authorities to change the place of work because of the pandemic, (6) access to free psychological counseling, (7) provision of guidelines for the use of telepsychiatry, and (8) provision of a dedicated closed platform for audiovisual communication in telemedicine.

## Conclusions

The COVID-19 pandemic has affected early-career psychiatrists in Europe to varying degrees. The results of the study point to areas where decision-makers can improve the learning and working conditions for ECPs to increase their resilience, which can help to better prepare for future outbreaks.

A shorter training, better adherence to COVID-19-related recommendations, and access to a dedicated platform for telemedicine predicted a higher confidence in knowledge on COVID-19 symptoms and management among ECPs. Providing specific recommendations during the COVID-19 pandemic, access to additional educational activities for ECPs, following COVID-19-related recommendations, and access to protective equipment were the significant predictors of a higher confidence in managing patients with comorbidity of COVID-19 and mental disorders.

Following COVID-19-related recommendations at the workplace and access to the dedicated platform for telemedicine predicted assessing the COVID-19 pandemic as less negative for well-being. On the other hand, additional educational activities for ECPs, lack of possibility to take the exam on time during the COVID-19 pandemic, better adherence to COVID-related recommendations, diagnosis of COVID-19, and access to free psychological counselling were predictors of a more positive assessment of the impact of supervisors and co-workers on ECPs’ well-being.

Several factors were associated with the use of telepsychiatry. The obligation to change the place of work predicted a decreased satisfaction with telepsychiatry as well as a decreased willingness to use telepsychiatry after the COVID-19 pandemic. In contrast, a diagnosis of COVID-19, having recommendations for telepsychiatry, and access to a dedicated platform for telemedicine were predictors of an increased satisfaction with telepsychiatry. Similarly, additional educational activities for ECPs, having recommendations for telepsychiatry, and access to the dedicated platform for telemedicine were predictors of a greater willingness to use telemedicine after the pandemic.

## Supporting information

Pinto da Costa et al. supplementary material Pinto da Costa et al. supplementary material

## Data Availability

Data supporting the findings of this study are available from the corresponding author TMG on request.

## Supplementary material

The supplementary material for this article can be found at http://doi.org/10.1017/S1092852925100734.
