# Supplementary material for: Early career psychiatrists in Europe during the COVID-19 pandemic: a cross-sectional study
Source: CNS Spectr. 2025 Nov 21;30(1):e98. doi: 10.1017/S1092852925100734 (PMC13064691; doi:10.1017/S1092852925100734)
Supplement: Pinto da Costa et al. supplementary material [file S1092852925100734sup001.pdf]

Early Career Psychiatrists in Europe during COVID-19 outbreak

The survey is conducted by the European Psychiatric Association Early Career Psychiatrists Committee (EPA ECPC) and the EPA ECPC Task Force on Meetings and Associations with the collaboration and support of the European Federation of Psychiatric Trainees (EFPT).

The aim of the survey is to investigate the impact of the COVID-19 pandemic on education, working conditions and wellbeing of Early Career Psychiatrists (ECPs) in Europe.

EPA ECPC defines an "Early Career Psychiatrist" as a psychiatric trainee or a psychiatrist under 40 years of age or a psychiatrist less than 5 years after specialty (including both adult and child and adolescent psychiatrists).

The survey includes 24 questions and should take about 5-7 minutes to complete. It can only be filled once by one person. The survey is completely anonymous and the participation in this study is voluntary. It is possible to interrupt filling in the survey at any point, in such a case no data will be collected. The collected responses will be aggregated and analyzed; the aggregated results will be used in a scientific publication. Individual responses will not be disclosed.

In case of any questions, please contact EPA ECPC: ecpc@europsy.net, chair of EPA ECPC, Mariana Pinto da Costa: mariana.pintodacosta@gmail.com, chair of EPA ECP Task Force on Meetings and Associations, Tomasz M. Gondek: gondektm@gmail.com or EFPT at: president@efpt.eu

EPA ECPC website: https://www.europsy.net/early-career-psychiatrists-committee
EFPT website: http://efpt.eu/

* Indicates required question

1. I hereby confirm I am 18 years old or older and I agree to participate in this study. I also confirm I am an Early Career Psychiatrist (psychiatric trainee OR psychiatrist under 40 years of age OR psychiatrist less than 5 years after specialty). I am aware the survey can only be completed once. *

Mark only one oval.

- Yes
- No

Socio-demographics

2. 1. Gender: *

Mark only one oval.

- Female
- Male
- Non-binary
- Do not want to disclose

3. 2. Country where you currently work: *

____

4. 3. Professional status: *

Mark only one oval.

- Trainee in general adult psychiatry
- Trainee in child and adolescent psychiatry
- Specialist in general adult psychiatry under 40 years of age or with less than 5 years of clinical practice after specialty
- Specialist in child and adolescent psychiatry under 40 years of age or with less than 5 years of clinical practice after specialty
- Other: _______________________________

5. 4. Main place of work during the COVID-19 pandemic: *

Mark only one oval.

- Inpatient psychiatric ward
- Outpatient psychiatric clinic or day ward
- Individual private psychiatric practice
- Inpatient COVID-19 ward
- Outpatient COVID-19 clinic
- Other: ____________________

COVID-19 Knowledge and Training

6. 5. How confident are you in your knowledge on COVID-19 symptoms and management? *

Mark only one oval.

- Very confident
- Confident
- Not confident nor unconfident
- Unconfident
- Very unconfident

7. 6. How confident are you in managing patients with a comorbidity of COVID-19 and a mental disorder? *

Mark only one oval.

- Very confident
- Confident
- Not confident nor unconfident
- Unconfident
- Very unconfident

8. 7. Have any specific recommendations for ECPs during the pandemic been introduced in your country? Please choose all applicable (or “No”): *

Tick all that apply.

- [ ] Yes, by the National/regional authorities
- [ ] Yes, by National Psychiatric Association
- [ ] Yes, by other organizations
- [ ] No

9. 8. Have additional educational activities (courses, workshops, local conferences) on COVID-19 been offered for ECPs in your country? Please choose all applicable (or “No”): *

Tick all that apply.

- [ ] Yes, general activities for all MDs
- [ ] Yes, specific activities dedicated to all psychiatrists
- [ ] Yes, specific activities dedicated to ECPs
- [ ] No

10. 9. Have obligatory local training and educational activities for ECPs (courses, workshops, local conferences) been replaced with online activities? *

Mark only one oval.

- ( ) Yes, all obligatory activities have been converted to online activities
- ( ) Yes, some but not all obligatory activities have been converted online activities
- ( ) No, they have been carried out as usual (in person)
- ( ) No, they have been canceled but no online alternative has been offered.

11. 10. Has COVID-19 pandemic affected the duration of your training? Please choose all options that may apply (or “Not applicable”):

Tick all that apply.

- [ ] Yes, it extended the duration of my training
- [ ] Yes, it shortened the duration of my training
- [ ] Yes, it prevented me from taking the specialist exam as planned
- [ ] No
- [ ] Not applicable (I have completed my training and exams before the COVID-19 outbreak)

Workplace conditions

12. 11. Have you been obliged by the authorities to change the place of work because of the pandemic? *

Mark only one oval.

- ( ) Yes
- ( ) No

13. 12. The physical distancing and other recommendations related to COVID-19 prevention were followed at my workplace: *

Mark only one oval.

- ( ) Strongly agree
- ( ) Agree
- ( ) Neither agree nor disagree
- ( ) Disagree
- ( ) Strongly Disagree
- ( ) Not applicable (e.g. I work alone)

14. 13. Have personal protective equipment been sufficiently provided for the ECPs * in your workplace? Choose all applicable (or "No"):

Tick all that apply.

- [ ] Yes, they have been provided by the medical facility (hospital/clinic/university)
- [ ] Yes, they have been provided by the local or national authorities
- [ ] Yes, they have been provided by non-governmental organisations
- [ ] Yes, they have been provided by private entities or other individuals
- [ ] No, I had to buy or prepare the protective equipment myself

15. 14. Did you have access to free COVID-19 tests at your place of work? *

Mark only one oval.

- ( ) Yes
- ( ) No

16. 15. Have you been tested for SARS-CoV-2? *

Mark only one oval.

- ( ) Yes, at least one test positive
- ( ) Yes, all tests negative
- ( ) Yes, awaiting the results of my first test
- ( ) No

17. 16. Have you been clinically diagnosed with COVID-19? *

Mark only one oval.

- ( ) Yes, I have recovered
- ( ) Yes, I still have COVID-19
- ( ) No

18. 17. Have you been quarantined? *

Mark only one oval.

- Yes, due to clinical diagnosis of COVID-19
- Yes, due to positive test for COVID-19
- Yes, due to potential exposure to SARS-CoV-2 but not confirmed with test
- Yes, due to return from abroad
- No

Wellbeing and support

19. 18. How did COVID-19 pandemic affect your wellbeing? *

Mark only one oval.

- Very positively (increased resilience, increased sense of importance of my work)
- Rather positively (increased resilience, increased sense of importance of my work)
- No significant impact
- Rather negatively (increased stress and burnout, health concerns)
- Very negatively (increased stress and burnout, health concerns)

20. 19. What was the impact of your supervisors and/or co-workers on your wellbeing? *

Mark only one oval.

- Very positive
- Rather positive
- No significant impact
- Rather negative
- Very negative
- Not applicable

21. 20. Did you have access to free psychological counseling? Choose all applicable (or “No”): *

Tick all that apply.

- [ ] Yes, provided/funded by the medical facility (hospital/clinic/university)
- [ ] Yes, provided/funded by the local or national authorities
- [ ] Yes, provided/funded by non-governmental organisations
- [ ] Yes, provided/funded by private entities or individual professionals
- [ ] No

Telepsychiatry

22. 21. Was telepsychiatry used in your country during the pandemic? *

Mark only one oval.

- ( ) Yes, there are recommendations how to proceed with telepsychiatry in my country
- ( ) Yes, however there are no recommendations how to proceed with telepsychiatry in my country
- ( ) No, it is not legally approved in my country

23. 22. What means of communication did you use for telepsychiatry? Please choose all options that may apply (or “Not applicable”): *

Tick all that apply.

- [ ] Dedicated closed platform for audiovisual communication in telemedicine
- [ ] General software for audiovisual communication (commonly used applications)
- [ ] Telephone or software for audio communication only
- [ ] Chat, text messages or e-mail
- [ ] Not applicable

24. 23. How satisfied are you with the use of telepsychiatry during the pandemic? *

Mark only one oval.

- Very satisfied
- Rather satisfied
- Moderately satisfied
- Rather dissatisfied
- Very dissatisfied
- Not applicable, never used telepsychiatry

25. 24. How likely are you to use telemedicine after the pandemic? *

Mark only one oval.

- Extremely likely
- Likely
- Neutral
- Unlikely
- Extremely unlikely
- Not applicable (e.g. no legal conditions for telemedicine)

This content is neither created nor endorsed by Google.

Google Forms
